# Empagliflozin add‐on therapy is superior to metformin monotherapy in diabetic patients with NAFLD: An open‐label, single‐center, pilot clinical trial

**DOI:** 10.1002/jgf2.723

**Published:** 2024-09-08

**Authors:** Ayda Esmaeili, Reza Pourahmad Azar, Mohammadreza Mohammad Hosseiniazar, Laya Hooshmand Gharabagh

**Affiliations:** ^1^ Department of Clinical Pharmacy, School of Pharmacy Urmia University of Medical Sciences Urmia Iran; ^2^ Experimental and Applied Pharmaceutical Sciences Research Center Urmia University of Medical Sciences Urmia Iran; ^3^ Student Research Committee, Urmia University of Medical Sciences Urmia Iran; ^4^ Department of Internal Medicine, School of Medicine Urmia University of Medical Sciences, Imam Khomeini Hospital Urmia Iran

**Keywords:** empagliflozin, fibroscan, metformin, non‐alcoholic fatty liver disease, type 2 diabetes

## Abstract

**Background:**

The prevalence of non‐alcoholic fatty liver disease (NAFLD), which is characterized by hepatic steatosis, inflammation, and advanced fibrosis, is high among type‐2 diabetes mellitus (T2DM) patients. Empagliflozin (EMPA), a sodium‐glucose cotransporter‐2 inhibitor, has been well established to improve glycemic status in T2DM. However, evidence of the desirable effects of EMPA, when added to the standard treatment in diabetics with coexisting NAFLD, has yet to be determined.

**Objective:**

The main objective of the current study is to explore the benefits of EMPA on hepatic fat content in patients with T2DM and NAFLD, who received metformin (MET) monotherapy.

**Methods:**

In this open‐label clinical trial study, 60 patients with T2DM and NAFLD were assigned to either the MET + EMPA or MET group in an up‐titrated manner for 24 weeks. Anthropometric characteristics, blood glucose indices, lipid profile, liver enzymes, and steatosis grades were measured at baseline and 24 weeks after the intervention.

**Results:**

The results showed that in patients with a mean age of 53.26 ± 7.64 who received MET+ EMPA, all the parameters had a greater decrease than the MET group. In addition, the reduction of FBS, BS, HbA1C, TG, and ALT had a statistically significant difference between the two groups after 24 weeks follow‐up (*p* < 0.05). Notably, in the MET+ EMPA group, there was a substantial improvement in steatosis grades based on the fibroscan and ultrasound modality results.

**Conclusion:**

The EMPA add‐on therapeutic schedule in uncontrolled T2DM patients with NAFLD significantly ameliorated steatosis stages, liver function, anthropometric features, and biochemical parameters.

## Graphical abstract

Empagliflozin add‐on therapy in uncontrolled T2DM patients with NAFLD significantly ameliorated steatosis stages, liver function, anthropometric features, and biochemical parameters.

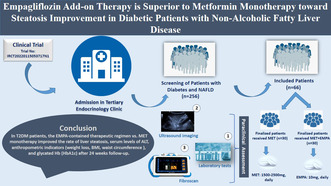

## 1 INTRODUCTION

The epidemiological analyses support either intricate or close relationships between non‐alcoholic fatty liver disease (NAFLD) and type 2 diabetes mellitus (T2DM) with a rapidly increasing incidence worldwide.^1^ In detail, NAFLD, defined as slowly progressed hepatic steatosis in >5% of hepatocytes,^2^ is an emerging chronic disease occurring in diabetic patients, with a high prevalence between 55% and 68% following the superimposable condition of insulin resistance (IR).^3^ An unfavorable lifestyle, obesity (BMI ≥30 kg/m^2^), dyslipidemia, T2DM, and other metabolic syndromes are involved in deriving this multifactorial condition.^4^ Notably, it has been well documented that individuals diagnosed with NAFLD have a twofold increased risk of T2DM.^5^, ^6^

Besides, those with T2DM co‐morbidity are at a higher risk of further steatohepatitis (NASH), advanced fibrosis, and related mortality.^7^ The early diagnosis and prime management of metabolic syndromes based on an appropriate therapeutic strategy are particularly important. In this regard, clinical scoring systems, for example, fibroscan, are useful tools in the early diagnosis of NAFLD and in predicting fibrosis. To date, rising trials are under development to find the optimal therapeutic schedule considering the liver function in T2DM‐NAFLD patients.^8^ A recent cohort study elegantly evaluated the potential variables involved in NAFLD occurrence in patients with T2DM. Intriguingly, the results showed that the probability of incident NAFLD in diabetic patients is directly associated with an increased body mass index (BMI) but not glycated hemoglobin. In addition, diabetic patients who were on sodium‐glucose cotransporter‐2 (SGLT2) inhibitors, glucagon‐like peptide‐1 receptor antagonists, and insulin experienced a lower incidence of NAFLD.^9^

Metformin, belonging to the biguanides class, is an extensively prescribed and well‐known anti‐diabetic medication, which has off‐labeled indications because of a wide array of benefits toward other metabolic syndromes, for example, NAFLD through AMPK‐dependent and independent manners.^10^, ^11^, ^12^

Empagliflozin (EMPA), a member of sodium‐glucose co‐transporter 2 inhibitors, is a new‐generation anti‐diabetic drug controlling glycemia by increasing urinary glucose excretion.^13^ It has been proven that EMPA has further cardiovascular and renal outcomes accompanied by positive metabolic impacts in favor of body weight loss and NAFLD improvement.^14^

## 2 OBJECTIVES

To the best of our knowledge, the application of a multi‐drug strategy with various modes of action would be more appreciable in clinical practice. In this regard, the primary study endpoint was to assess the synergistic potential of the EMPA (10 mg, once daily) + MET (1500–2500 mg, daily) versus MET monotherapy in diabetic patients with NAFLD.

## 3 METHODS

### 3.1 Study population

**FIGURE 1:**
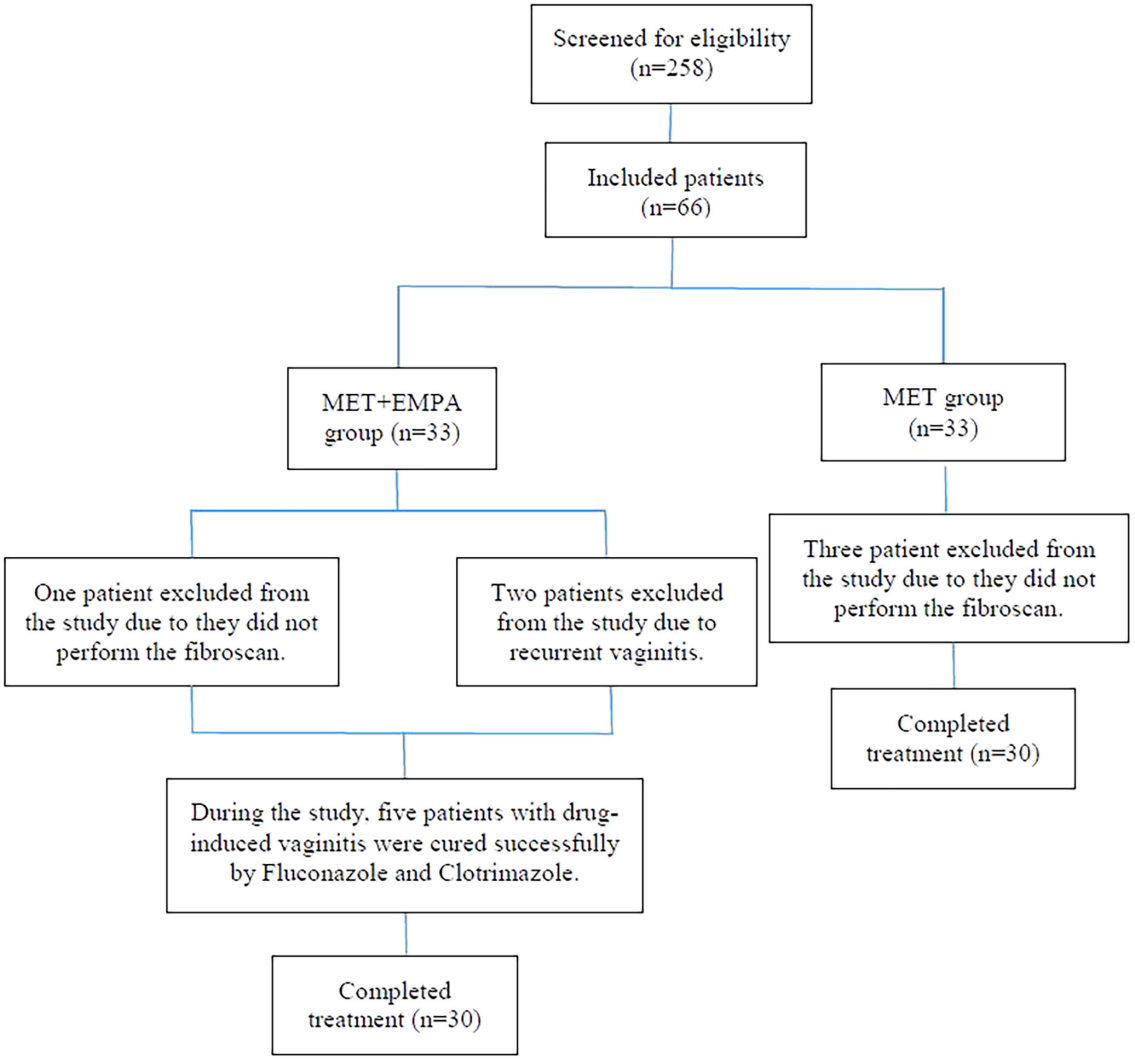
The Diagram of the Patients Screening.

In this single‐center, open‐label, prospective, observational pilot clinical trial, all SGLT2i‐naïve and MET‐treated T2DM outpatients with diagnosed NAFLD, aged 18 years and over, blood glucose (HbA1C = 6.5%–10%), BMI >20 kg/m^2^, and weight change <10% in the last 3 months were included from July 2022 to April 2023. All enrolled patients had at least grade 1 of ultrasound and fibroscan examinations. The exclusion criteria were other types of diabetes, treatment with other anti‐diabetic drugs, renal dysfunction estimated glomerular filtration (eGFR) <45 mL/min/1.73m^2^ at the beginning of the study, history of pancreatitis or diseases related to the pancreas, ALT more than twice normal, and heart failure defined as New York Heart Association (NYHA) class III or IV. In addition, autoimmune hepatitis and viral hepatitis, a history of alcohol consumption (>140 mL for women and >250 mL for men per week), and lactating/pregnant women were also considered to be excluded from the study. Finally, a total of 60 eligible patients with metformin‐treated T2DM who attended the tertiary endocrinology clinic were enrolled in the present study and followed up for 24 weeks. Patients who were assigned to a MET monotherapy group with dose escalation (from 1500 up to 2500 mg/day) or those with higher Hb A1C initiated the combination therapy of MET + EMPA according to the standards of care introduced by the American Diabetes Association (ADA). The diagram of the study population screening has been detailed in Figure 1.

### 3.2 Liver assessment and paraclinical procedures

All patients were examined by liver ultrasound imaging and FibroScan®, and the liver stiffness measurement (LSM) score was determined through the transient elastography technique. Based on previous studies, a 10% improvement and reduction in LSM score was defined as a response to treatment.^15^, ^16^

During the study, the measurement of lipid profile, including low‐density lipoprotein (LDL), high‐density lipoprotein (HDL), total cholesterol (Chol), and triglyceride (TG), functional enzymes of the liver such as alkaline phosphatase (ALP), alanine transaminase (ALT), and aspartate aminotransferase (AST) was performed. Furthermore, anthropometric characteristics (weight, waist circumference, and BMI), and other biochemical parameters including blood glucose (BG), fasting plasma glucose (FPG), glycated hemoglobin (HbA1C), and serum creatinine (Cr) were assessed in serum samples collected at baseline and after 24 weeks.

### 3.3 Intervention

#### 3.3.1 Patients were assigned into two groups

The control group was given MET as the standard treatment (initiating dose <1500 mg, 3months before the onset of the study, increased to a maximum dose of 2500 mg during 24 weeks if needed). The intervention group received EMPA (10 mg, once daily, obtained from Abidi pharmaceutical company, Iran) add‐on to the standard of care (patients who received 1500 mg MET for 3 months before the onset of the study). All patients were followed up at 24 weeks. To modulate the lifestyle, all participants were required to perform moderate‐intensity physical activity according to the metabolic equivalent task (METS) at least three times per week along with given standard dietary advice.^17^ The patients were monthly followed through a phone call and were consulted when some adverse effects were observed.

### 3.4 Data processing and statistical analysis

According to the formula, the sample size was calculated as 60 subjects with a power of 80%, 5% significance, and 20% dropout rate.^18^

Continuous variables are reported as mean ± SD, while categorical data are expressed as numbers and percentages. The normal distribution of the data was evaluated using the Kolmogorov–Smirnov test. The student's *t*‐test was used to compare the mean of continuous variables with normal distribution, while the Mann–Whitney *U*‐test was used if they were not normal. The Chi‐squared test was also used to analyze qualitative data. The ANCOVA test was applied to assess the significant differences of variables at the baseline. Data analysis was performed using SPSS version 17 software and the significance level was considered <0.05.

### 3.5 Ethics approval and consent to participate

The ethical approval for this research was issued from our institution [Ethics Committee Review (2021)]. Written informed consent was provided by all participants for further data recording and analysis. This study was performed in accordance with the ethical standards as laid down in the 1964 Declaration of Helsinki and its later amendments or comparable ethical standards. Of note, the information of all patients was confidential, and no additional cost was incurred to them. Also, our study adheres to CONSORT guidelines.

## 4 RESULTS

This pilot study enrolled 60 patients with a confirmed NAFLD (mean age of 53.26 ± 7.64; 33 women, 55%). The baseline characteristics of all patients were detailed in Table S1. The comparison of the assessed variables at baseline and 24 weeks after the intervention in each group has been brought in Table 1. Our findings showed that in the control group, the assessed variables significantly ameliorated when compared to baseline except for BMI, HDL, ALT, and Cr (*p* < 0.05). However, in the MET + EMPA group, all variables had a significant improvement except for Cr (Table 1, *p* < 0.05). The results of fibroscan and ultrasound analysis noted that there was no significant difference between the two groups in the case of fatty liver degree before the intervention.

**TABLE 1:** The comparison of the mean of the studied variables before and after the intervention in each group.

Variables	MET with dose escalation			EMPA add‐on		
	Baseline	After 24 weeks	*p* Value	Baseline	After 24 weeks	*p* Value^a^
Weight (kg)	86.0 ± 13.27	84.62 ± 12.73	0.001	86.78 ± 12.9	81.0 ± 10.85	<0.001
Waist circumference (cm)	105.2 ± 10.16	104.43 ± 9.84	0.007	106.83 ± 7.18	102.4 ± 6.25	<0.001
BMI (kg/m^2^)	29.97 ± 3.23	29.39 ± 3.05	0.87	31.23 ± 4.29	29.22 ± 4.02	**<0.001**
Systolic pressure	129.3 ± 17.74	118.4 ± 16.59	0.012	117.83 ± 9.35	110.37 ± 15.16	0.027^b^
Diastolic pressure	83.53 ± 11.15	72.47 ± 16.59	<0.001	78.5 ± 6.84	68.93 ± 10.38	<0.001
FPG (mg/dL)	150.8 ± 30.01	122.63 ± 20.29	<0.001	190.43 ± 52.24	118.53 ± 21.85	<0.001
BG	218 ± 50.13	166.87 ± 47.7	<0.001	274.2 ± 76.44	179.57 ± 32.02	<0.001
HbA1C	7.96 ± 0.99	7.18 ± 0.49	<0.001	8.73 ± 1.25	6.89 ± 0.56	<0.001
TG (mg/dL)	179.3 ± 68.95	141.73 ± 44.88	<0.001	256.77 ± 141.51	158.4 ± 69.45	0.001
Chol (mg/dL)	195.8 ± 54.67	142.57 ± 45.55	<0.001	205.17 ± 61.37	129.27 ± 38.41	<0.001
LDL (mg/dL)	112.6 ± 28.26	83.27 ± 20.84	<0.001	107.73 ± 35.59	73.77 ± 21.12	<0.001
HDL (mg/dL)	42.6 ± 9.89	44.37 ± 7.05	0.2	44.53 ± 10.84	49.85 ± 7.88	**0.027** ^c^
AST (IU/L)	22.33 ± 9.69	18.7 ± 8.24	<0.001	22.9 ± 13.15	14.85 ± 6.07	0.001^c^
ALT (IU/L)	27.13 ± 10.78	25.0 ± 9.72	0.16	27.6 ± 11.82	19.26 ± 7.36	**0.002** ^c^
ALP (IU/L)	160.3 ± 62.58	114.93 ± 32.82	<0.001	177.33 ± 52.8	135.5 ± 36.55	0.001^c^
Cr (mg/dL)	0.89 ± 0.18	0.92 ± 0.16	0.51	0.91 ± 0.18	1.1 ± 0.24	0.4^c^

^a^ *p* Value less than 0.05 was considered significant.

^b^ The Wilcoxon test was used.

^c^ The paired *t*‐test was used.

As shown in Table 2, in the MET group it has been shown that there was no significant difference regarding the fibroscan and LSM after the intervention (*p* = 0.50), while a relative improvement was observed in the ultrasound imaging (Table 2, *p* < 0.008). While, in the MET + EMPA group, a remarkable improvement in steatosis grades was detected based on fibroscan, LSM score, and ultrasound examination (Table 2, *p* ≤ 0.001). In patients who received combination therapy, all studied biochemical parameters (except for HDL, LDL, Chol, ALP, and Cr) had a greater reduction compared to patients who received MET only. We also could not find a remarkable difference in terms of hemodynamic status (systolic/diastolic pressure, Table 3, *p* > 0.05).

**TABLE 2:** Comparing the steatosis grades before and after 24 weeks of the intervention in each group.

Fatty liver degree	MET with dose escalation (%)^a^			EMPA add‐on^a^	
		Baseline	After 24 weeks	Baseline	After 24 weeks
Fibro scan	0–1	18 (60)	20 (66.7)	15 (50)	29 (96.7)
	2–3	12 (40)	10 (33.3)	15 (50)	1 (3.3)
*p*‐Value		0.5		**0.001**	
Ultrasound	0–1	10 (33.3)	18 (60)	11 (36.7)	28 (93.3)
	2–3	20 (66.7)	12 (40)	19 (63.3)	2 (6.7)
*p*‐Value		**0.008**		**<0.001**	
LSM (kPa)		7.02 ± 1.12	6.95 ± 1.02	7.23 ± 1.09	5.92 ± 0.89
*p*‐Value		0.5		**0.001**	

Abbreviations: EMPA, Empagliflozin; KPa, kilopascals; LSM, Liver Stiffness Measurement; MET, Metformin.

^a^ Data was reported as *n* (%).

**TABLE 3:** Comparison of the mean changes of the studied variables between the two groups.

Variables	MET with dose escalation	EMPA add‐on	*p* Value^a^
Weight (kg)	−1.38 ± 2.12	−5.78 ± 3.6	<0.001
Waist circumference (cm)	−0.77 ± 0.26	−4.4 ± 2.39	<0.001
BMI (kg/m^2^)	−0.57 ± 0.7	−2.00 ± 1.02	<0.001
Systolic pressure (mmHg)	−10.9 ± 4.04	−7.46 ± 3.19	0.51^b^
Diastolic pressure (mmHg)	−11.07 ± 2.47	−9.57 ± 1.8	0.63^b^
FPG (mg/dL)	−28.17 ± 15.15	−71.9 ± 21.43	<0.001
BG	−94.63 ± 54.87	−51.37 ± 28.1	0.001
HbA1C (%)	−0.78 ± 0.7	−1.84 ± 1.2	<0.001^b^
TG (mg/dL)	−37.57 ± 9.37	−98.37 ± 25.78	0.031^b^
Chol (mg/dL)	−53.23 ± 32.2	−75.9 ± 46.02	0.08
LDL (mg/dL)	−29.33 ± 21.7	−33.97 ± 25.2	0.45
HDL (mg/dL)	1.77 ± 0.44	4.67 ± 1.9	0.23
AST (IU/L)	−3.6 ± 2.9	−8.05 ± 1.18	0.064
ALT (IU/L)	−2.13 ± 1.05	−8.34 ± 3.38	0.034
ALP (IU/L)	−45.37 ± 6.91	−41.83 ± 6.58	0.83
Cr (mg/dL)	0.02 ± 0.1	0.2 ± 0.3	0.46

^a^ *p* Value less than 0.05 was considered significant.

^b^ Adjustments have been made compared to the base values.

Notably, the results of fibroscan, ultrasound, and LSM assessment after 24 weeks indicated a significant improvement of hepatic steatosis in the case group when compared with the control counterpart (fibroscan: grades 0–1 increased to 40%, and grades 2–3 decreased to 3.3%, *p* = 0.001; ultrasound: grades 0–1 increased to 50%, and grades 2–3 decreased to 6.7%) (Table 4, *p* < 0.001).

**TABLE 4:** Comparing the steatosis grades between the two groups after 24 weeks.

Fatty liver assessment		MET with dose escalation (%)^a^	EMPA add‐on	*p* Value^b^
Fibroscan	0	2 (6.7)	12 (40)	0.001
	1	18 (60)	17 (56.7)	
	2	10 (33.3)	1 (3.3)	
Ultrasound	0	3 (10)	15 (50)	<0.001
	1	15 (50)	13 (43.3)	
	2	12 (40)	2 (6.7)	
LSM (kPa)		6.94 ± 1.01	5.92 ± 0.89	0.001

Abbreviations: EMPA, Empagliflozin; KPa, kilopascals; LSM, Liver Stiffness Measurement; MET, Metformin.

^a^ Data are reported as *n* (%).

^b^ *p* Value less than 0.05 was considered significant.

## 5 DISCUSSION

To date, it has been well established that NAFLD and T2DM are common metabolic conditions leading to pertinent adverse outcomes.^19^ A gold standard for NAFLD management is mainly referred to regulating metabolic risk factors, for example, good glycemic control (HbA1C ≤7%) and BMI. On the other hand, the current therapeutic options in patients with co‐existent NAFLD and T2DM are still limited. Beyond the liver gluconeogenesis induced by MET, it has been revealed that an additional protective effect against hepatic steatosis and IR can be exerted through the transcription factor EB‐autophagy pathway.^20^ However, long‐term use of MET, in turn, has the potential to increase the risk of NAFLD development in diabetic patients.^21^ Given the existing limited data in favor of the efficacy of SGLT2 inhibitors such as EMPA add‐on to the standard of care for the improvement of the NAFLD parameters in T2DM patients, we sought to evaluate the potential impacts of combination therapy of two anti‐diabetic medications (MET and EMPA) with a distinct mode of action in uncontrolled diabetic patients with NAFLD who were on a moderate‐to‐high statin.

Noticeably, the findings of this study indicated that in patients who received MET + EMPA, the anthropometric characteristics, ALT, blood glucose indices (FPG, BG, and HbA1C), and the lipid profile had a greater improvement when compared to the MET counterpart. The fibroscan results also displayed a significant retrieving in steatosis grade in the case group. Regarding the serum levels of HDL, although a greater increase was found in the EMPA group, the differences were not significant between the two groups. One reason to explain the positive impact of EMPA on anthropometric characteristics is referred to enhance glucose excretion through the urine because of the subsequent SGLT‐2 inhibitors‐induced glycosuria, leading to a dynamic change in the body fluid composition that may contribute to weight loss and increased lipid consumption.

In terms of NAFLD, the available evidence also revealed that SGL2 inhibitors exert some advantages through multiple mechanisms. In the experimental setting, it has been shown that EMPA, by polarizing M2 macrophages, has the potential to modulate energy expenditure, inflammatory response (decreased TNFα levels and obesity‐induced chronic inflammation), and IR.^22^ Also, EMPA can shift energy metabolism toward lipid consumption, enhance AMP‐activated protein kinases, as well as acetyl‐CoA carboxylase phosphorylation in the skeletal muscle, and further increase hepatic and serum levels of fibroblast growth factor 21. Moreover, EMPA has a key role in the expression of uncoupling protein 1 (UCP1) in the adipose tissues,^22^, ^23^ and consequently promotes weight loss and augments liver function.

A recent clinical study (the IMAGIN pilot study) conducted in Italy has also highlighted the beneficial effects of combination therapy with EMPA against NAFLD progression in diabetic patients.^24^ In parallel with our findings, the authors reported a substantial improvement in terms of BMI, HbA1C, ALT, and steatosis degree in comparison to baseline.^24^ However, some limitations noted in the IMAGIN study should be taken into account: the dose used for both MET and EMPA and the increased trend of dosing have not been stated. In addition, the favorable changes in lipid profile, weight, and waist circumference were not investigated.

A recent publication also indicated a superior impact of EMPA compared to pioglitazone (thiazolidinedione drug class) against liver steatosis and fibrosis in diabetic patients with NAFLD.^17^ Regarding the EMPA role in lipid modulation, Liu et al. proposed that EMPA has a protective potential against atherosclerotic events by affecting lipid profile and sympathetic activity in the experimental model.^25^ In contrast, the results of a meta‐analysis conducted to evaluate the effects of EMPA on lipid profile in diabetes did not show any benefits in this era.^26^ Although the positive effect of EMPA on lipid profile is controversial, we elegantly showed a desirable trend in serum levels of lipid indices in the EMPA+MET‐treated group. In line with our findings, Kuchay et al. using MRI‐derived proton density fat fraction also demonstrated that EMPA can decline the liver fat content and ameliorate the levels of liver enzymes in uncontrolled diabetic patients who received standard treatment.^27^

Besides, in the present study, the primary hemodynamic parameters were assessed, and a remarkable difference was not observed between the two groups consistent with Kuchay et al. study.^27^ While Lai et al. revealed that the positive impact of EMPA in diabetic patients with nonalcoholic steatohepatitis is able to significantly improve the hemodynamic parameters, as well.^28^

## 6 CONCLUSION

In T2DM patients, the EMPA‐contained therapeutic regimen versus MET monotherapy significantly ameliorated the rate of liver steatosis, serum levels of ALT, anthropometric indicators, and glycated Hb only after 24 weeks of follow‐up. The preliminary results of this pilot study need to be confirmed in further large‐scale and multi‐center research.

## 7 STUDY LIMITATION

The present study has some limitations. The first one refers to the small sample size because of the emerging COVID‐19 pandemic, which remarkably influenced the admission rate of diabetic outpatients, particularly in referral medical centers. Moreover, the lack of some patients' collaboration during the study follow‐up was taken into account as another reason for the study limitation. Also, it would be better to document any lifestyle changes such as dietary habits and physical activity made by individuals, particularly in the case group.

## AUTHOR CONTRIBUTIONS

AE and LHG: designed and conceptualized the study, contributed to data collection, and interpretation and writing the manuscript; RPA: performed the process; MMH: collected and analyzed the data and screened the articles; LHG: drafted the manuscript. All authors contributed to the manuscript, and read and approved the final manuscript.

## FUNDING INFORMATION

None.

## CONFLICT OF INTEREST STATEMENT

The authors have stated explicitly that there are no conflicts of interest in connection with this article.

## ETHICS STATEMENT

Ethics approval statement: The protocol of this study was approved by the medical ethics committee of our institution.

Patient consent statement: Informed consent was obtained from the participants.

Clinical trial registration: None.

## Supporting information


Table S1.

## Data Availability

All data and material collected during this study are available from the corresponding author upon reasonable request.
