# Supplementary material for: Empagliflozin add‐on therapy is superior to metformin monotherapy in diabetic patients with NAFLD: An open‐label, single‐center, pilot clinical trial
Source: J Gen Fam Med. 2024 Sep 8;25(6):351–7. doi: 10.1002/jgf2.723 (PMC11565072; doi:10.1002/jgf2.723)
Supplement: Supplementary file 1 — Table S1. [file JGF2-25-351-s001.docx]

**Supplementary Table 1.** The Baseline Characteristics

| Variables  Mean Age (yr) | | 53.26 ±7.64 |  |  |
| --- | --- | --- | --- | --- |
| MET (%) MET+EMPA (%) P value | | | | |
| Gender | ***Male*** | 14 (46.7) | 13 (43.3) | 0.79 |
|  | ***Female*** | 16 (53.3) | 17 (56.7) |  |
| Weight | | 86.0±13.27 | 86.78±12.9 | 0.82^§^ |
| Waist circumference | | 105.2±10.16 | 106.83±7.18 | 0.47^§^ |
| BMI (kg/m2) | | 29.97±3.22 | 31.23±4.29 | 0.2^§^ |
| Systolic Pressure (mmHg) | | 129.3±17.74 | 117.83±9.35 | 0.003^£^ |
| Diastolic Pressure (mmHg) | | 83.53±11.15 | 78.5±6.84 | 0.039 |
| FPG (mg/dl) | | 150.8±30.01 | 190.43±52.24 | 0.001^§^ |
| BG | | 218±50.13 | 274.2±76.44 | 0.001^§^ |
| HbA1C (%) | | 7.96±0.99 | 8.73±1.25 | 0.011^§^ |
| TG (mg/dl) | | 179.3±68.95 | 256.77±141.51 | 0.01^£^ |
| Chol (mg/dl) | | 195.8±54.67 | 205.17±61.37 | 0.53^§^ |
| LDL (mg/dl) | | 112.6±28.26 | 107.73±35.59 | 0.56^§^ |
| HDL (mg/dl) | | 42.6±9.89 | 44.53±10.84 | 0.47^§^ |
| AST (IU/L) | | 22.33±9.69 | 22.9±13.15 | 0.85^§^ |
| ALT (IU/L) | | 27.13±10.78 | 27.6±11.82 | 0.87^§^ |
| ALP (IU/L) | | 160.3±62.58 | 177.33±52.8 | 0.26^§^ |
| Cr (mg/dL) | | 0.89±0.18 | 0.91±0.18 | 0.78^§^ |

^§^ Using the Independent t-test.

^£^ Using the Mann-Whitney U test.
